# Percutaneous Interventions for Heart Failure in Congenital Heart Disease

**DOI:** 10.1007/s11886-026-02371-7

**Published:** 2026-04-16

**Authors:** Lore Schrutka, Raymond N. Haddad, Enrico Piccinelli, Thuso David, Murat Sürücü, Shakeel A. Qureshi

**Affiliations:** 1 Department of Internal Medicine II, Division of Cardiology, Medical University of Vienna, Vienna, Austria; 2 Filière des Cardiopathies Congénitales Enfant Adultes, Hôpital Marie Lannelongue, Centre Constitutif du Réseau Maladies Rares Malformations Cardiaques Congénitales Complexes - M3C, Hôpitaux Saint Joseph et Marie Lannelongue, Le Plessis Robinson, France; 3 Faculté de Médecine Kremlin-Bicêtre, Université Paris-Saclay, Le Kremlin- Bicêtre, France; 4 Hypertension Pulmonaire: Physiopathologie et Innovation Thérapeutique (HPPIT), Université Paris-Saclay, INSERM, UMR_S1358, Hôpital Marie Lannelongue, Le Plessis-Robinson, France; 5 Cardiology, Cardiac Surgery and Heart Lung Transplantation, ERN GUARD HEART, Bambino Gesù Hospital and Research Institute, IRCCS, Rome, Italy; 6 Department of Paediatrics and Adolescent Health, University of Botswana, Gaborone, Botswana; 7 Department of Pediatric Cardiology, Dr. Siyami Ersek Thoracic and Cardiovascular Surgery Training and Research Hospital, Istanbul, Turkey; 8 Department of Paediatric Cardiology, Evelina London Children’s Hospital, Guy’s and St Thomas’ NHS Foundation Trust, London, SE1 7EH UK; 9 Direction de la Recherche et de l’Innovation Médicale (DRIM), Marie Lannelongue Innovation Center (MALIC), Hôpitaux Saint Joseph Marie Lannelongue, Le Plessis-Robinson, France

**Keywords:** Congenital heart disease, Heart failure, Invasive monitoring, Percutaneous interventions, Mechanical circulatory support, Fontan circulation

## Abstract

**Purpose of Review:**

Heart failure (HF) is a major cause of morbidity and mortality in patients with congenital heart disease (CHD), driven by residual lesions and progressive ventricular dysfunction. The heterogeneity of CHD anatomy and physiology often limits the applicability of standard HF therapies. This review aims to summarize contemporary catheter-based strategies used to diagnose, stabilize, and treat HF in patients with CHD across both acute and chronic clinical settings, and to evaluate their role in improving outcomes and delaying surgical or transplant-based therapies.

**Recent Findings:**

Recent advances in transcatheter technology have expanded therapeutic options for HF in CHD. Invasive hemodynamic monitoring and implantable sensors enable more precise assessment and remote management of complex circulations. Short-term mechanical circulatory support systems, including extracorporeal membrane oxygenation and percutaneous ventricular assist devices, are increasingly used as bridges to recovery or transplantation. Catheter-based closure of shunts (e.g., atrial or ventricular septal defects and patent ductus arteriosus) and creation of controlled shunts using devices such as atrial flow regulators provide strategies to optimize hemodynamics. Transcatheter valve therapies, including pulmonary valve implantation and atrioventricular valve repair, have become important alternatives to repeat surgery. In patients with failing Fontan circulation, interventions such as fenestration creation, pathway stenting, collateral embolization, and emerging lymphatic procedures have demonstrated promising clinical benefits.

**Summary:**

Percutaneous interventions have become central to the management of HF in CHD, offering minimally invasive, anatomy-specific approaches that can stabilize acute decompensation, address residual lesions, and palliate chronic circulatory failure. These strategies enable individualized treatment pathways and may delay or reduce the need for surgical reintervention or transplantation. Continued technological innovation, improved patient selection, and multidisciplinary expertise will be essential to further refine these approaches.

## Introduction

Congenital heart disease (CHD) survivors represent a rapidly growing population, many of whom will develop heart failure (HF) over time due to their residual lesions, prior surgical interventions, or progressive myocardial dysfunction. The rate of hospitalisation for patients with CHD is twice that of the general population, with 50% of patients being hospitalized and 16% requiring intensive care [1, 2]. Although only around 7% of hospitalisations are due to HF, [1] it represents the leading cause of death among CHD patients [3, 4]. The underlying anatomical and physiological heterogeneity, particularly in patients with complex or palliated CHD such as Fontan circulation or systemic right ventricles, complicates standard HF management. Recent advances in percutaneous interventions have transformed the therapeutic landscape for acute and chronic HF in CHD patients by offering less invasive, adaptable strategies that stabilize patients, improve hemodynamics, and delay or avoid the need for surgery or transplantation. This review provides a comprehensive synthesis of catheter-based strategies across the continuum of HF in CHD, including invasive hemodynamic monitoring, mechanical circulatory support, closure and creation of shunts, valve interventions, and novel approaches for failing Fontan physiology. Additionally, the review highlights the indications, techniques, and outcomes of these therapies in the growing population of patients with CHD and HF.

## Acute Heart Failure in Congenital Heart Disease

Acute heart failure (AHF) is a complex clinical syndrome characterized by the sudden onset or worsening of HF signs and symptoms, requiring urgent medical attention. In patients with CHD, AHF can be precipitated by structural deterioration, arrhythmias, residual shunts, or elevated pulmonary arterial pressures. Accurate and timely hemodynamic assessment is essential for the effective management of AHF in patients with CHD, as assessment can be particularly challenging in this population due to complex anatomies and variable pathophysiology [5, 6]. Invasive hemodynamic monitoring informs therapeutic decisions and facilitates rapid stabilization [7]. It also provides the basis for decisions regarding the need for mechanical circulatory support or urgent catheter-based interventions [8].

### Invasive Hemodynamic Monitoring in Acute Heart Failure

AHF arises from a multifactorial process involving myocardial dysfunction, volume overload, and neurohormonal activation. Clinical examination and ultrasound often fall short in defining the precise hemodynamic profile needed for individualized therapy [8]. Invasive monitoring offers direct assessment of intracardiac pressures, cardiac output, and vascular resistances, guiding tailored interventions, particularly in complex congenital heart disease or challenging postoperative settings where non-invasive data may be inconclusive [8, 9].

#### Common Invasive Monitoring Techniques

Right and left heart catheterization remain the definitive tools for hemodynamic assessment in AHF and complex CHD. Right heart catheterization (RHC) provides evaluation of volume status, pulmonary artery pressures, cardiac output, pulmonary vascular resistance (PVR), whilst left heart catheterization (LHC) is useful for assessing systemic ventricular pressures, outflow tract obstruction, or coronary arterial abnormalities [9]. Combined RHC and LHC offer comprehensive mapping of systemic and pulmonary circulations, vital in CHD with systemic right ventricles, single ventricles, or significant shunt lesions [8, 9].

#### Pulmonary Artery Catheter

The pulmonary artery catheter (PAC, or Swan-Ganz) allows continuous monitoring of filling pressures, cardiac output, and mixed venous oxygen saturation (ScvO₂). In CHD, PAC is primarily used during diagnostic or interventional catheterization, particularly when non-invasive methods are insufficient for evaluating PVR and shunt physiology [9, 10]. It plays a pivotal role in managing refractory AHF, especially when initiating mechanical circulatory support such as extracorporeal membrane oxygenation (ECMO) or ventricular assist devices (VAD). Routine PAC use remains controversial; even in adult patients with HF, the ESCAPE trial demonstrated no survival benefit, [11] and robust pediatric data remain lacking.

#### Central Venous Pressure Monitoring

Central venous pressure (CVP), measured via a central line, reflects right atrial pressure and provides a rough estimate of intravascular volume. Its clinical value increases when paired with dynamic indices or ScvO₂, offering indirect insight into global perfusion and oxygen balance [12].

#### Invasive Systemic Arterial Pressure Monitoring

Invasive arterial lines enable continuous systemic blood pressure monitoring, essential for titrating vasoactive medications, guiding mechanical circulatory support, and performing frequent arterial blood gas analyses in intensive care unit (ICU) or catheterization settings. However, there is a growing tendency to favor non-invasive alternatives to reduce the risk of vascular complications associated with arterial cannulation [13, 14].

#### Advanced Hemodynamic Monitoring

Techniques such as Pulse index Continuous Cardiac Output (PiCCO) which combines transpulmonary thermodilution with pulse contour analysis, and thoracic bioimpedance offer less invasive alternatives but have limited validation in CHD, especially in the presence of intracardiac shunts [15].

#### Clinical Utility

Invasive hemodynamic monitoring improves diagnosis by distinguishing cardiogenic shock, detecting pulmonary hypertension, and clarifying etiology. Continuous assessment of systemic arterial pressures and cardiac output supports real-time treatment adjustment and decisions on escalation or de-escalation of care [8, 16]. However, procedural risks limit its use to selected intensive care patients, particularly in pediatric and congenital heart disease populations, where non-invasive approaches are increasingly preferred [17, 18].

### Invasive Mechanical Circulatory Support (ECMO, Impella, Venting Strategies)

Mechanical circulatory support (MCS), particularly short-term percutaneous options, has emerged as a critical therapeutic bridge in this population - serving roles from stabilization to decision-making, recovery, or transition to durable support or transplantation and in some cases also as chronic or “destination” therapy [19–21]. The 2022 registry report from the International Society for Heart and Lung Transplantation (ISHLT) shows that more than a third of recipients of transplants for CHD were supported by MCS, reflecting its increasing use [22].

#### Short-term Support

##### ECMO in Congenital Heart Disease

Veno-arterial ECMO (VA-ECMO) remains the most widely utilized short-term MCS in CHD populations [19]. Its utility spans acute decompensation, postoperative low cardiac output syndrome, and extracorporeal cardiopulmonary resuscitation (ECPR) [19]. Early initiation (within six hours) is linked to better outcomes [23, 24], though complications such as bleeding, thrombosis, stroke, and infection remain common [25, 26]. Cannula placement in CHD can be challenging, and unique problems such as steal from the systemic circulation by Blalock–Taussig shunts or aortopulmonary collaterals can limit full cardiac unloading.

##### The Impella^®^ Device

The Impella^®^ (Abiomed, MA, USA) is a percutaneous catheter-mounted micro-axial pump that provides partial circulatory support by unloading the left ventricle. Impella use in CHD is limited by anatomical and size constraints, but it has shown feasibility in selected larger children and adolescents with systemic left ventricular dysfunction [27, 28]. Right-sided mechanical circulatory support systems are not used as much, the applications of the Impella RP^®^, have been reported in cases such as Ebstein anomaly [29].

Combined use of Impella and ECMO (“ECMELLA”) can mitigate ECMO-induced afterload, thus improving pulmonary congestion and facilitating myocardial recovery but carries increased risks of vascular injury, device migration, hemolysis, and thromboembolism [30, 31].

##### Venting Strategies During ECMO

Left ventricular distension during VA-ECMO is associated with worse outcomes and may require venting [30]. Venting options include passive strategies, such as atrial septostomy (percutaneous or surgical), implantation of Atrial Flow Regulator (AFR) devices to decompress the left atrium and active strategies, such as left atrial or LV cannulation, either surgically or via transseptal approach and Impella-assisted unloading in ECMELLA configurations [30, 32]. Indications include persistent pulmonary edema, elevated filling pressures, absent aortic valve opening, or intracardiac stasis [30].

#### Durable Support Options

Durable ventricular assist devices are increasingly used in CHD as bridges to transplantation, with outcomes approaching those of non-CHD patients when implanted in experienced centers [33]. However, patients with systemic right ventricles or Fontan circulation present unique technical and physiological challenges, underscoring the need for individualized strategies [13, 34–38].

## Chronic Heart Failure

Chronic HF (CHF) is becoming more common among patients with CHD due to improved survival rates and the long-term effects of anatomical and physiological changes. This condition can present as systemic right ventricular failure, sub-pulmonary or single ventricle dysfunction, valve regurgitation, or Fontan-associated circulatory failure. Unlike acquired cardiomyopathies, standard HF therapies can vary in effectiveness, necessitating an individualized, anatomy-specific approach. Symptoms such as exercise intolerance, edema, and hepatic congestion may develop insidiously and often require detailed imaging and hemodynamic assessment for interpretation. New strategies, including remote monitoring, transcatheter valve and shunt interventions, and lymphatic therapy for Fontan insufficiency, offer new opportunities to delay disease progression. Long-term care in specialized centers is essential for optimizing outcomes in this heterogeneous and vulnerable patient population.

### Invasive Monitoring of Chronic Heart Failure (Cardiomems)

The CardioMEMS HF System^®^ (Abbott, Abbott Park, IL, USA), a wireless pulmonary artery pressure sensor, is approved for use in adults with HF and has been applied off-label in selected CHD populations [39].

Possible indications for invasive monitoring of device implantation are single ventricle patients palliated with Fontan circulation, patients with d-transposition of the great arteries (d-TGA) after atrial switch, ccTGA and a borderline systemic ventricle. Continuous pressure data provide insight into daily life hemodynamics, avoiding confounding effects of anesthesia, mechanical ventilation, or fasting associated with catheterization [40–43]. Remote monitoring may be particularly valuable for patients living far from specialized centers.

Evidence for reduction in HF hospitalizations or mortality in CHD remains limited and inconsistent [41]. Reduced adherence to data transmission has been a major limiting factor [43, 44]. Additionally, sensor recalibration during follow-up has been required more frequently in CHD than in acquired HF, with reported pressure discrepancies of several mmHg [41, 45].

Procedural challenges arise from complex native or post-surgical anatomy, especially in Fontan patients, in whom long sheaths and prior pulmonary artery stents may complicate device placement. Reported complications include device migration, embolization, and pulmonary artery distortion [41, 45]. Thromboembolic risk appears low but may be increased in Fontan physiology, necessitating careful anticoagulation management [41, 45].

Despite these limitations, CardioMEMS may reduce the need for surveillance catheterization and allow remote titration of medical therapy in carefully selected patients. Optimization of patient education, caregiver involvement, and multidisciplinary monitoring may enhance adherence and maximize potential benefit [46].

### Shunt Closure Procedures for HF (ASD, SVASD, VSD, PDA, Fistulas)

Shunt lesions, including atrial septal defects (ASDs), ventricular septal defects (VSDs), patent ductus arteriosus (PDA), and arteriovenous fistulas impose chronic volume overload, chamber dilation, pulmonary hypertension, and eventual HF. Percutaneous closure has become the preferred approach for most of these lesions [47].

#### Atrial Septal Defect Closure

ASD closure is indicated when significant left-to-right shunting results in right heart volume overload [48]. Elective closure is recommended between the ages of three and five [48]. Although ASDs are usually asymptomatic in childhood, some infants may present with HF, respiratory distress, or failure to thrive, which warrants earlier intervention. In adults, age-related changes, such as reduced left ventricular compliance, can exacerbate shunting and make ASDs symptomatic later in life [48–50]. In older patients or those with diastolic dysfunction, closure must be approached cautiously, as elimination of the shunt may precipitate pulmonary edema [51]. Balloon test occlusion and fenestrated devices may help identify and mitigate this risk [52, 53].

Transcatheter ASD closure, introduced in 1974 and revolutionized by the Amplatzer Septal Occluder (ASO), is now standard practice [54, 55]. Selection of occluder devices depends on the defect size, its location and rim adequacy, with self-centering occluders generally favored for most cases, whilst non-self-centering devices are being used for small or eccentrically located defects [56, 57].

#### Superior Sinus Venosus Defect Closure

Superior sinus venosus ASDs are typically associated with partial anomalous pulmonary venous drainage of the right upper pulmonary vein into the SVC or the right atrium [58]. Due to the complexity of the anatomy, surgical repair has traditionally been the standard treatment.

In 2014, a novel transcatheter technique using a covered stent in the SVC to redirect pulmonary venous return emerged as a viable option in selected cases [59, 60]. Careful patient selection, aided by multimodality imaging and superior vena cava (SVC) balloon interrogation, is essential for procedural success [61–65].

#### Ventricular Septal Defect Closure

Ventricular septal defects (VSD) closure is indicated for hemodynamically significant defects with acceptable pulmonary vascular resistance [66, 67]. Muscular and selected perimembranous VSDs are amenable to percutaneous closure, though the latter carry a higher risk of heart block [68, 69]. Device embolization, valvular regurgitation, and arrhythmias are rare but recognized complications that have decreased with improved technology and techniques [70, 71].

#### Patent Ductus Arteriosus Closure

Patent ductus arteriosus (PDA) closure is indicated in symptomatic patients with left heart overload and is routinely performed using occluder devices or coils, even in small infants [58, 72, 73]. Devices such as the Amplatzer Duct Occluder (ADO) and its variants (ADO II, ADO II AS), which offer versatility to accommodate different duct morphologies and patient size and can be used in small infants under echocardiographic guidance [74–76]. Coils may occasionally be used for small PDAs [77]. Potential complications include device embolization, hemolysis, stenosis of the left pulmonary artery and vascular obstruction, especially in small infants [77, 78].

#### Fistulas

*Coronary-cameral fistula*, most often involving the right coronary artery and the right atrium or ventricle and occasionally the pulmonary artery warrant intervention with significant left-to-right shunting, evidence of myocardial ischemia or progressive enlargement [79]. Transcatheter closure is first-line in suitable patients, with surgery reserved for infants or complex anatomies [80].

*Systemic arteriovenous fistulas* can lead to high-output cardiac states with right heart volume overload, with the preferred treatment being embolization, using coils, liquid agents such as Onyx, focal vessel occlusion for major aortopulmonary collaterals or surgical techniques, depending on lesion complexity [81].

### Creation of Shunts for Heart Failure (Fenestrated Atrial Devices, Transcatheter Potts shunt, Enlargement of restrictive ASD, Enlargement of VSD)

In selected patients with advanced HF, controlled shunt creation may provide hemodynamic benefits by decompressing high-pressure chambers or improving preload. Techniques include atrial septostomy, implantation of fenestrated atrial devices, and creation of systemic-to-pulmonary shunts.

#### The Atrial Flow Regulator

The Atrial Flow Regulator (AFR) (Occlutech Holding AG, Schaffhausen, Switzerland) is a self-expandable fenestrated device designed to create a calibrated interatrial shunt. Although approved for HF with preserved or reduced ejection fraction [82, 83], it has been used off-label in CHD, pulmonary hypertension, as a venting strategy during ECMO in children with end-stage cardiomyopathies and is considered effective and safe in creating a stable fenestration in Fontan circulation [32, 42, 84–89].

The device offers predictable fenestration size, high patency rates, and technical feasibility even in small children [42, 85]. In mid-term follow-up, patency is high (~ 91%), with minimal thromboembolic complications, even in right-to-left shunt settings [89].

#### ASD Creation

Percutaneous ASD creation or enlargement is crucial for palliation or as a bridge to definitive therapies in cases of transposition of the great arteries, and restrictive atrial septum in tricuspid atresia or hypoplastic left heart syndrome [9, 90, 91]. Techniques include the Rashkind balloon atrial septostomy, static balloon dilation, blade septostomy, radiofrequency perforation, atrial septal stenting or AFR implantation, depending on anatomy and age [9].

#### Transcatheter Creation of Potts Shunt

The transcatheter Potts shunt recreates a surgical anastomosis between the left pulmonary artery and descending aorta and is used as palliation in refractory suprasystemic pulmonary arterial hypertension [92, 93]. Early reports show improvement in functional class and right ventricular unloading, but procedural risks and high reintervention rates limit widespread use [93–95]. Long-term durability remains a challenge [96]. Despite its limitations, the transcatheter Potts shunt marks a significant advance in palliative care for patients who are not eligible for surgical shunt creation.

#### Percutaneous Valve Interventions for Heart Failure

Valvar dysfunction is a major contributor to HF in CHD [47]. Percutaneous valve interventions offer effective alternatives to surgery, particularly in high-risk patients or those requiring repeat interventions [97].

#### Pulmonary Valve Interventions

*Balloon Pulmonary Valvuloplasty*(BPV) remains the first-line treatment for isolated congenital pulmonary valve stenosis in all age groups [47, 98], providing durable relief of obstruction and low reintervention rates [99, 100]. A balloon-to-annulus ratio of 1.2 to 1.25 is considered more effective than larger ratios, achieving comparable gradient reductions whilst reducing the incidence of pulmonary regurgitation [101, 102].

Since its introduction in 2000 [103], balloon-expandable *Transcatheter Pulmonary Valve Replacement*(tPVR) has become the standard alternative to surgical valve replacement in dysfunctional conduits or bioprosthetic valves [104], and is increasingly applied in post-transannular patch anatomies using larger self-expanding valves [105–107]. The advantages of tPVR include a reduced need for surgical reintervention and improved hemodynamics, however, the risk of prosthetic valve endocarditis remains a major concern [108].

#### Aortic Valve Interventions

*Balloon aortic valvuloplasty* (BAV) is the preferred initial intervention for congenital aortic stenosis, particularly in neonates and children [109]. Although effective acutely, progressive aortic regurgitation often necessitates later surgical valve replacement [110]. Whilst BAV remains the preferred initial treatment due to its less invasive nature, recent data suggest that surgical valvotomy may reduce the need for reintervention, especially if tri-leaflet valve morphology can be achieved following repair [111].

Although *Transcatheter Aortic Valve Replacement*(TAVR) has been extensively studied in adults with calcific AS [112], its use in children and patients with CHD is limited by anatomical challenges and the prevalence of aortic regurgitation or mixed valve disease [113]. Early case reports and small case series have demonstrated the off-label use of modified devices, such as the Melody and Sapien and other valves, in selected patients with CHD [114, 115

#### Atrioventricular Valve Interventions

Atrioventricular (AV) valve regurgitation significantly contributes to HF, particularly in patients with systemic right ventricles and Fontan circulation. *Transcatheter Edge-to-Edge Repair* (TEER) techniques such as the MitraClip™ (Abbott Laboratories, IL, USA) and *Valve-in-Valve Procedures* in failing bioprosthetic AV valves have been performed in selected high-risk patients and have demonstrated symptomatic and hemodynamic improvement [116, 117,118 These interventions require meticulous planning and multidisciplinary expertise.

### Balloon Angioplasty and Stenting of Coarctation of the Aorta

Coarctation of the aorta increases ventricular afterload, which can ultimately result in HF [119]. Management options include surgical repair, balloon angioplasty, and stent implantation, with choice guided by patient age, anatomy, and prior interventions [120].

*Balloon Angioplasty* is widely accepted for recoarctation and for native coarctation in infants and small children, particularly when vessel size limits stent placement [121]. Whilst effective, balloon angioplasty is associated with higher rates of elastic recoil, restenosis, and vascular injury, especially in older patients [122].

*Endovascular Stenting* has become first-line therapy for coarctation in adolescents and adults, offering superior gradient reduction and lower rates of restenosis compared with balloon angioplasty alone [119, 121]. Both covered and bare-metal stents demonstrate similar efficacy, with covered stents preferred when there is concern for vessel injury [121]. Advances in stent technology have enabled treatment in increasingly smaller patients, including as a bridge to definitive surgery in neonates [123].

Successful intervention is defined by reduction of the gradient across coarctation to less than 10 mmHg, which is associated with improved long-term outcomes [124]. Lifelong surveillance is required to monitor for restenosis, aneurysm formation, and systemic hypertension.

### Transcatheter Interventions in the Failing Fontan Circulation

Fontan failure is characterized by systemic venous congestion, low cardiac output, and lymphatic dysfunction leading to exercise intolerance, and multi-organ complications [125, 126]. Transcatheter interventions are central to the management and aim to optimize circuit geometry, reduce venous pressures, and palliate symptoms. Transcatheter interventions have emerged as critical tools to preserve function, palliate symptoms, and delay the need for transplant [126, 127].

#### Fenestration and Transcatheter Offloading in the Failing Fontan

Transcatheter Offloading in the Failing Fontan provides a controlled right-to-left shunt that lowers central venous pressure and improves preload [125–127]. Techniques vary by Fontan type and include transseptal puncture, radiofrequency perforation combined with stent implantation, and off-label use of the fenestrated AFR device [89, 128, 129]. Whilst effective, these interventions carry a risk of thrombosis and require long-term antithrombotic therapy and surveillance [89, 128, 130].

#### Embolization of Aortopulmonary and Veno-venous Collaterals

*Embolization of Aortopulmonary Collaterals* reduces ventricular volume overload, whilst *Embolization of Veno-Venous Collaterals* may improve oxygenation in selected patients [125, 126, 131, 132]. Hemodynamic assessment is essential, as indiscriminate closure may worsen Fontan pressures. Nevertheless, embolization with microcoils and vascular plugs, often yields immediate improvements, but high rates of collateral recurrence demand long-term surveillance [125, 126].

#### Fontan Conduit and Pathway Stenting

Stenting of stenotic Fontan pathways has become a vital method to restore conduit patency and improve hemodynamic [133]. Balloon-expandable covered stents are the primary choice for treating stenoses at key sites, including the extracardiac conduit, pulmonary arteries, and veno-atrial junctions [126, 127]. Immediate results consistently show marked pressure gradient reduction and improved cardiac output, often resulting in symptomatic improvement. 133–135 In pediatric patients, somatic growth demands staged redilation [133]. Nonetheless, restenosis from neointimal proliferation and stent fractures remain significant long-term challenges [136]. Advanced imaging modalities, such as high-resolution MRI and 3D rotational angiography, combined with computational fluid dynamics, are transforming procedural planning [137, 138]. Bioresorbable scaffolds and drug-eluting stents hold promise in reducing restenosis rates but currently lack the necessary diameters and radial force required for Fontan circuit applications [139].

#### Emerging Transcatheter Therapies and Future Perspectives

*Emerging Lymphatic Interventions*, including thoracic duct embolization and decompression techniques, have shown promise in treating protein-losing enteropathy and plastic bronchitis [140, 141]. One such technique, the innominate vein turn-down, creates a direct connection to the atrium, facilitating decompression and improving lymphatic flow in selected patients with failing Fontan physiology [142]. These therapies represent an important advance but should be performed in highly specialized centers.

*Transcatheter Edge-to‐Edge Repair* (TEER) for AV valve regurgitation, is emerging as a viable adjunct in managing Fontan failure, though currently, only small case series are available [118].

## Conclusions

**Fig. 1:**
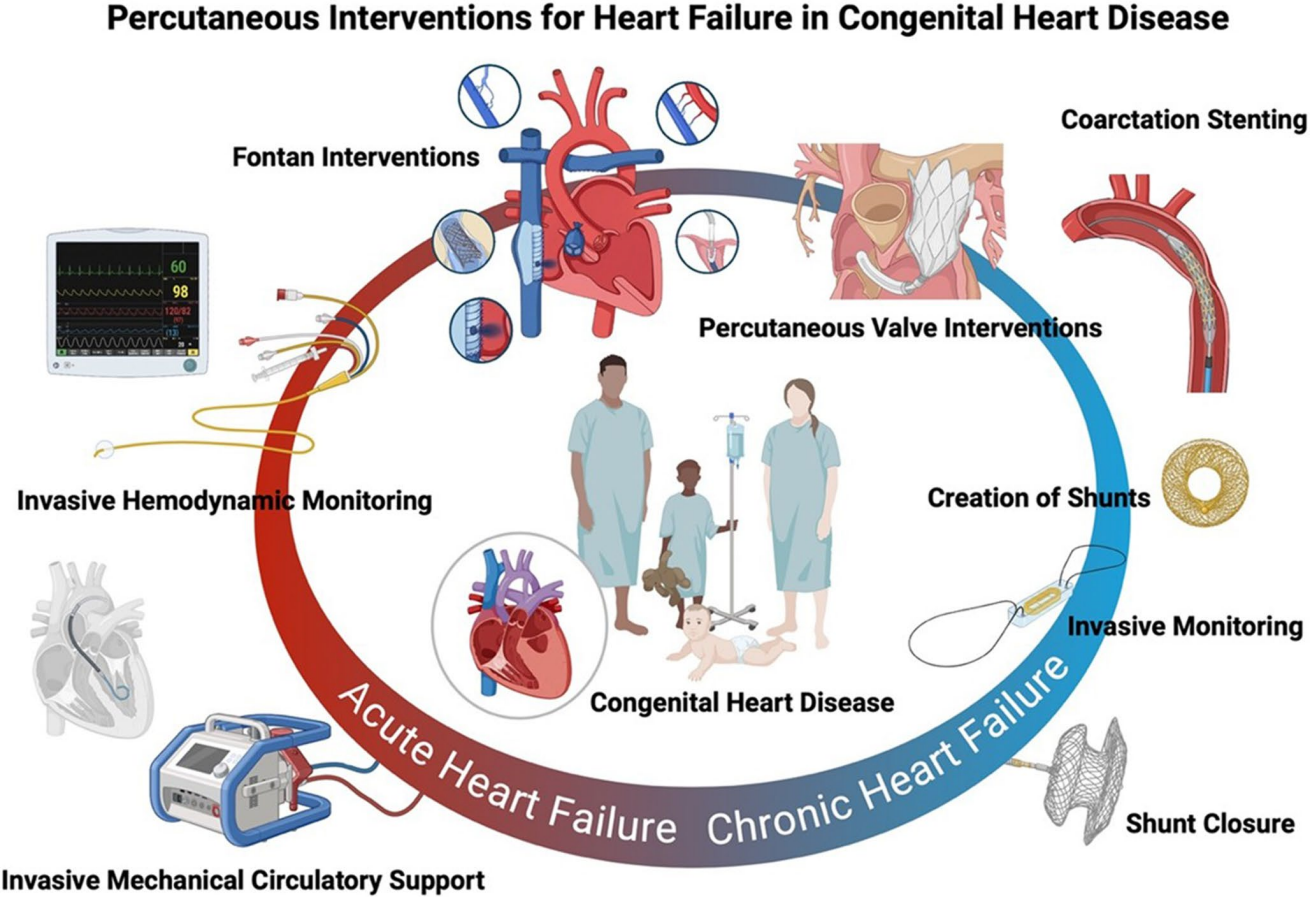
Percutaneous strategies are applied across the spectrum of acute and chronic heart failure in congenital heart disease, including invasive monitoring, mechanical support, shunt manipulation, valve and vascular interventions, and Fontan-specific therapies

Percutaneous interventions have become increasingly integral to the management of HF in patients with CHD, offering minimally invasive, lesion-specific solutions across the spectrum of acute and chronic presentations (Fig. 1). In acute decompensation, invasive hemodynamic monitoring guides decision-making, whilst short-term mechanical support systems, such as ECMO and percutaneous ventricular assist devices serve as critical bridges to recovery or transplant. Chronic management strategies include remote pressure monitoring with CardioMEMS and fenestration devices such as the AFR, tailored to unload systemic or pulmonary circulations. Closure of hemodynamically significant shunts (ASD, VSD, PDA) reduces volume overload, whilst percutaneous valve implantation and repair offer durable options for valvar dysfunction. In the failing Fontan circulation, catheter-based fenestration, stenting, collateral embolization, and emerging lymphatic interventions have shown promise in alleviating symptoms and prolonging survival. As procedural techniques and device platforms continue to evolve and expertise deepens, percutaneous strategies for HF in CHD will continue to grow under longitudinal surveillance.

## Key References

- Rosenthal E, Qureshi SA, Sivakumar K, et al. Covered Stent Correction for Sinus Venosus Atrial Septal Defects, an Emerging Alternative to Surgical Repair: Results of an International Registry. Circulation. 2025;151(11):744–756. https://doi.org/10.1161/circulationaha.124.070271. 
  This international registry provides important contemporary evidence supporting transcatheter techniques for the correction of superior sinus venosus atrial septal defects. The study demonstrates high procedural success and favorable safety outcomes, highlighting a minimally invasive alternative to surgical repair in selected patients.
- Goldstein BH, McElhinney DB, Gillespie MJ, et al. Early Outcomes From a Multicenter Transcatheter Self-Expanding Pulmonary Valve Replacement Registry. J Am Coll Cardiol. 2024;83(14):1310–1321.
  This multicenter registry reports early outcomes with newer self-expanding transcatheter pulmonary valve systems. The findings support the growing role of transcatheter pulmonary valve replacement as an effective alternative to repeat surgery in patients with right ventricular outflow tract dysfunction related to congenital heart disease.
- Flores-Umanzor E, Luna-López R, Cepas-Guillen P, et al. Transcatheter Interventions in Adults With Fontan Palliation. Circ Cardiovasc Interv. 2024;17(12):e014699. https://doi.org/10.1161/circinterventions.124.014699.
  This contemporary review outlines the expanding role of transcatheter interventions in adults with Fontan circulation, including fenestration creation, pathway stenting, and collateral embolization. It provides important insight into catheter-based strategies aimed at improving hemodynamics and delaying advanced heart failure therapies in this complex population.

## Data Availability

No datasets were generated or analysed during the current study.

## Abbreviations

AA: Ascending Aorta
ACC: American College of Cardiology
ACTION: Advanced Cardiac Therapies Improving Outcomes Network
ADO: Amplatzer Duct Occluder
AFR: Atrial Flow Regulator
AHA: American Heart Association
AHF: Acute Heart Failure
APC: Aortopulmonary Collateral
ASD: Atrial Septal Defect
ASO: Amplatzer Septal Occluder
AV: Atrioventricular
AVVR: Atrioventricular Valve Regurgitation
BAV: Balloon Aortic Valvuloplasty
CHD: Congenital Heart Disease
CHF: Congestive Heart Failure
CVP: Central Venous Pressure
ECMO: Extracorporeal Membrane Oxygenation
ECPR: Extracorporeal Cardiopulmonary Resuscitation
ESC: European Society of Cardiology
FDA: Food and Drug Administration
HF: Heart Failure
HFSA: Heart Failure Society of America
HLHS: Hypoplastic Left Heart Syndrome
ICU: Intensive Care Unit
INTERMACS: Interagency Registry for Mechanically Assisted Circulatory Support
ISHLT: International Society for Heart and Lung Transplantation
LV: Left Ventricle
LVAD: Left Ventricular Assist Device
MRI: Magnetic Resonance Imaging
NYHA: New York Heart Association
PAH: Pulmonary Arterial Hypertension
PB: Plastic Bronchitis
PDA: Patent Ductus Arteriosus
PLE: Protein-Losing Enteropathy
PS: Pulmonary Stenosis
PVR: Pulmonary Vascular Resistance
RV: Right Ventricle
RVOT: Right Ventricular Outflow Tract
SCAI: Society for Cardiovascular Angiography and Interventions
SV: Systemic Ventricle
SVASD: Superior Sinus Venosus Atrial Septal Defect
SVC: Superior Vena Cava
TAVR: Transcatheter Aortic Valve Replacement
TGA: Transposition of the Great Arteries
TPS: Transcatheter Potts Shunt
VAD: Ventricular Assist Device
VSD: Ventricular Septal Defect
VVC: Venovenous Collateral
